# Fatigue is a pain—the use of novel neurophysiological techniques to understand the fatigue-pain relationship

**DOI:** 10.3389/fphys.2013.00104

**Published:** 2013-05-13

**Authors:** Alexis R. Mauger

**Affiliations:** Endurance Research Group, School of Sport and Exercise Sciences, University of KentChatham, Kent, UK

Fatigue can be defined as any exercise-induced reduction in the ability to exert muscle force or power, regardless of whether or not the task can be sustained (Gandevia, 2001). There is no doubt that in sports performance where time to completion is the outcome measure, the management of fatigue is probably the determining factor of success. However, in exercise physiology, interventions and mechanisms have traditionally be measured using time to exhaustion tasks, where “the point of fatigue” occurs at task failure. The problem with this approach is that in sports performance, tasks are self-paced, and therefore in a successful performance, task failure (and thus the traditional concept of fatigue) never actually occurs. Therefore, which mechanisms govern fatigue in task failure, and which mechanisms govern work rate regulation (and thus the management of the process of fatigue) are very different constructs, which should not be used synonymously.

Whilst fatigue has previously been explained from a peripheral perspective (Kent-Braun, 1999), this standpoint is largely based on fatigue at task failure. More recently, greater focus has been placed on central mechanisms of fatigue (Gandevia, 2001; Noakes, 2012), so that now the role of the brain as a contributor to fatigue (at least in self-paced performance) is largely uncontested. This has led to the suggestion that perceived exertion is primarily derived from central command (Marcora, 2009), and conversely that somatosensory feedback from locomotor muscles is the major cause of fatigue (Amann and Secher, 2010). However, it is more likely that fatigue (in terms of endurance performance), is balanced through an interaction of these efferent and afferent systems (Perrey, 2010). By attributing a more “intelligent” role for the brain, Noakes' (2012) theory of central control suggests that afferent feedback from the periphery is collated and processed in conjunction with past experience and current knowledge of the external environment to produce a “sensation” of fatigue on which the level of motor output is based. The conjecture that fatigue is a sensation is probably not compatible with its role in task failure, and is therefore a highly disputed concept. However, that decision-making during (and before) exercise is based on the knowledge of the task and the self (i.e., pacing), and that this is a major factor in endurance performance (Mauger et al., 2009), is more widely accepted. Therefore, the variables which are used in this decision-making process (i.e., to up- or down-regulate work rate) are of considerable interest to the physiologist and psychologist, as the moderation of these has the potential to improve athletic performance.

There are numerous studies which have provided insight into which factors influence decision-making in exercise performance, including but not limited to; distance knowledge (Mauger et al., 2009), prior experience (Mauger et al., 2009), distance feedback (Albertus et al., 2005; Mauger et al., 2010b), performance feedback (Mauger et al., 2011), knowledge of ambient conditions (Castle et al., 2012), knowledge of the physiological state of the body (Tucker et al., 2004; Rauch et al., 2005; Noakes and Marino, 2007), external competition (Corbett et al., 2012) and motivation (Cabanac, 1986). Whilst these studies provide a useful insight into what information is required to successfully regulate work rate and which aspects may improve this process, many of these variables are either inherently present (or not) during competition, or are very difficult to manage. However, one variable which is frequently referred to by athletes, coaches and commentators, but has received peculiarly little focus in research, is exercise-induced pain. Indeed, pain during exercise is popularly referred to as a major inhibiting factor during exercise, and it is suggested that athletes who are better able to tolerate or overcome pain will be more successful. As exercise-induced pain is present in nearly all forms of time-dependent competition, the requirement for understanding its influence on performance and how athletes respond to its presence is imperative. The International Association for the Study of Pain define pain as an unpleasant sensory and emotional experience associated with actual or potential tissue damage, or described in terms of such damage. This implies that pain is always subjective, has an emotional element and that this is not always directly related to the magnitude of the nociceptive signal. Therefore, the nature of the athlete's perception of the nociceptive signal is a key element to the experience of pain, which provides a promising basis for effective intervention and subsequent performance improvement.

During high intensity exercise, Type III and IV nociceptors, are stimulated by mechanical pressure, heat, cold, noxious pressure, and endogenous pain producing (algesic) substances. These contribute to the acute muscle pain associated with particular forms of exercise (O'Connor and Cook, 1999), which is ultimately interpreted in the brain and perceived as exercise-induced pain. Given that the conscious awareness of the self is an important facet in pacing based decisions in exercise, it is logical that exercise-induced pain may provide an individual with useful information regarding the relative “strain” on the body, and thus use this to inform a conscious decision to increase or decrease exercise intensity (Mauger et al., 2010a). If this is the case, then exercise-induced pain may be a likely contender contributing to “fatigue” (or the management of fatigue) in exercise performance.

Despite this apparent link, limited research has been conducted on the fatiguing effects of exercise-induced pain (Mauger et al., 2010a; Mauger and Hopker, 2012). Those studies which have investigated the role of pain on exercise have produced equivocal results—Khan et al. (2011) demonstrated that deep muscle pain, elicited by the injection of hypertonic saline, reduced maximal voluntary torque (~5%) but had no effect of voluntary activation or submaximal torque output. Conversely, Hollander et al. (2008) found that partial occlusion increased pain and rating of perceived exertion (RPE) for a given exercise intensity. Ray and Carter (2007) and Hudson et al. (2008) found that the analgesics codeine and aspirin did not effect performance of fixed intensity exercise, whilst Mauger et al. (2010a) found that acetaminophen significantly improved self-paced exercise performance. Additionally, the competitive opioid naloxone appears to increase RPE and reduce time to exhaustion (Sgherza et al., 2002) and chronic pain affects spatial perceptions in a similar manner to fatigue, so that target distances appear further away than they really are (Witt et al., 2009). Of these studies, only one (Mauger et al., 2010a) study provides insight into the role of pain in self-paced performance, where the decision to up- or down-regulate work rate is likely to be most strongly influenced by pain perception. Mauger et al. (2010a), found that 1.5 g of acetaminophen allowed well-trained cyclists to produce a higher power output for a given perceived pain and RPE, which resulted in a significantly faster time to complete a 16.1 km cycle time trial. The authors concluded that participants were willing to engage in a given level of pain, and that they regulated this by moderating their power output.

Studies investigating the influence of exercise-induced pain on performance can be approached by either increasing or decreasing the pain experienced during exercise. Perhaps part of the difficulty in designing and conducting these type of studies is finding interventions which accurately replicate the type of pain experienced during intense physical exercise, or that moderate pain without effecting other physiological functions which may also effect exercise capacity. Techniques to induce pain may range from cold or heat, direct electric current, tonically induced pain, mechanical pain and topically induced pain [for more detail on these methods, the Reader is referred to the excellent review by Staahl and Drewes (2004)]. Understanding the pain pathways involved with each of these techniques is important because as type III and IV afferents are not only involved in transmitting a nociceptive signal, but are also involved in moderating the exercise pressor reflex, it is difficult to attribute changes in performance to differences in pain perception or cardiovascular control—increased pain may stimulate heart rate and minute ventilation for example. Conversely, reducing pain during exercise creates its own inherent problems and confounding variables which may alter exercise capacity through mechanisms other than analgesia [e.g., epidural–motor output (Amann et al., 2009); fentanyl—exercise pressor response (Amann et al., 2008); acetaminophen—cortico-spinal excitability (Mauger and Hopker, 2013); aspirin—coagulation, inflammation (Hudson et al., 2008)]. These issues are further compounded by the fact that techniques used for brain imaging [functional magnetic resonance imaging, electroencephalography (EEG), positron emission tomography, functional near infrared spectroscopy (fNIRS)] present several issues both with data acquisition during exercise, and differentiating differences in brain activity between exercise and the consequential pain. Accordingly, novel physiological techniques which are capable of inducing pain altering effects, and monitoring other indirect responses are of particular interest to the exercise scientist.

As the sensory and emotional aspects of pain ensure that it is ultimately a subjective experience (Rainville, 2002) that may be modulated via a number of factors outside of the internal transmission system, modulation of pain through brain based interventions are particularly attractive. Indeed, whilst the sensory aspects of pain are transmitted to the somatosensory (VPL, VPM) thalamic nuclei to cortical areas (S1, S2 and the posterior parietal cortex), and subsequently, from these areas to cortical limbic structures (insular cortex, anterior cingulate cortex), the emotional aspects of pain primarily involve the limbic system (Rainville, 2002). As such, the location of the areas where the brain processes the sensory and affective dimensions of pain are anatomically distinct, which means that the targeting of these areas to specifically moderate pain may provide an effective intervention.

Transcranial direct current stimulation (tDCS) is a non-invasive, transient type of neuro-stimulation which has successfully been used to treat stoke patients (Norris et al., 2010), depression (Boggio et al., 2007) and improve brain function (Marshall et al., 2004). This technique has also been shown to reduce both chronic and acute pain in healthy individuals and clinical populations (Boggio et al., 2008). Indeed, the use of tDCS to moderate pain appears to be particularly successful (Mylius et al., 2012b; Reidler et al., 2012), and can even be specifically targeted to brain regions so that modulation of emotional (Boggio et al., 2009) and sensory pain (Boggio et al., 2008) can be distinguished. The non-invasive, transient and target specific nature of a tDCS intervention provides a unique and exciting methodology by which the role of pain on exercise performance can be robustly tested. Some exercise physiology research groups have already started using this technique to investigate its potential to improve performance (Cogiamanian et al., 2007; Tanaka et al., 2009; Okano et al., 2013). Whilst improvements in exercise capacity or muscular force have been demonstrated, the limited experimental design of these studies has allowed little insight into the mechanisms underpinning the apparent effect, nor have the studies used a self-paced model of exercise performance. Whilst the effect of tDCS on specific neural mechanisms may be difficult to measure, relatively simple measures such as manipulation checks on pain response (i.e., pain threshold tests) motivation (motivation assessed questionnaires) and parasympathetic activity (resting heart rate, blood pressure and gas analysis) would significantly strengthen experimental design. Additionally, where laboratories and research groups have access to more specialized equipment, further mechanistic measures should also be sought to be acquired. As tDCS has been shown to increase brain blood flow (Zheng et al., 2011) and increase cortical excitability (Nitsche and Paulus, 2001), fNIRS and transcranial magnetic stimulation (TMS) could be used to monitor these potential neurophysiological responses. Furthermore, whilst tDCS can be applied at rest, EEG may be used to identify whether activity has increased in the desired (or an undesired) region. In terms of using a tDCS intervention, researchers should be aware that there are several different tDCS set-ups (e.g., changing anodal or cathodal stimulation, brain area targeted and stimulation amplitude) which can be used to bring about different effects [for an excellent review the Reader is referred to Mylius et al. (2012a)]. Whilst anodal stimulation is generally accepted to increase excitability of the targeted region, and cathodal stimulation to decrease excitability (Nitsche and Paulus, 2001), the analgesic effects of these respective set-ups may be different depending on the brain region targeted. Stimulation (both anodal and cathodal) of the motor cortex (M1) is more consistent with increasing pain threshold (Boggio et al., 2008), whereas stimulation of the dorsolateral prefrontal cortex (DLPFC) appears to preferentially increase pain tolerance (Boggio et al., 2009). There is no agreed set-up for which to decrease exercise-induced pain, although it is likely that either (or both) anodal stimulation of the M1 and DLPFC will reduce the pain felt during intense exercise.

Thus, it appears that whilst no single technique may provide a “magic bullet” to examine the role of the brain (and exercise-induced pain) in exercise performance, a range of complimentary and novel techniques could be used to directly test a given mechanism (e.g., exercise-induced pain) in a reproducible and controlled manner. The challenge for research groups is now to forge collaborations of expertise, so that as many potential mechanistic explanations for an observed effect can be accounted for. With this in mind, perhaps well-known expressions such as “the man who can drive himself further once the effort gets painful is the man who will win,” can be accurately and robustly tested.
